# Impact of balanced versus unbalanced fluid resuscitation on clinical outcomes in critically ill children: protocol for a systematic review and meta-analysis

**DOI:** 10.1186/s13643-019-1109-2

**Published:** 2019-08-05

**Authors:** Anab Rebecca Lehr, Soha Rached-d’Astous, Melissa Parker, Lauralyn McIntyre, Margaret Sampson, Jemila Hamid, Kusum Menon

**Affiliations:** 1 Division of Critical Care, Department of Pediatrics, University of Ottawa, Children’s Hospital of Eastern Ontario, 401 Smyth Road, Ottawa, ON K1H 8L1 Canada; 2 Division of Emergency Medicine, Department of Pediatrics, University of Toronto, Hospital for Sick Children, Toronto, ON Canada; 3 Division of Critical Care, Department of Pediatrics, McMaster University, Hamilton, ON Canada; 4 Division of Critical Care, Department of Medicine, Ottawa Hospital Research Institute, University of Ottawa, Ottawa, ON Canada; 5 Library Services, Children’s Hospital of Eastern Ontario, Ottawa, ON Canada; 6 Clinical Research Unit, Children’s Hospital of Eastern Ontario, Ottawa, ON Canada

**Keywords:** Critically ill children, Resuscitation, Fluid bolus therapy, Isotonic fluid, Crystalloid fluid, Normal saline, Balanced fluid, Ringer’s lactate

## Abstract

**Background:**

Isotonic crystalloid fluid bolus therapy is used in critically ill children to restore or maintain hemodynamic stability. However, the ideal choice of crystalloid remains to be determined. The most easily available and most frequently used crystalloid is 0.9% saline, an unbalanced crystalloid, that has been associated with hyperchloremic metabolic acidosis and acute kidney injury (AKI). Balanced fluids such as Ringer’s lactate (RL) were developed to be closer to the composition of serum. However, they are more expensive and less readily available than 0.9% saline. Few trials have found RL to be associated with more favorable outcomes, but pediatric data is limited and inconsistent. The objective of the present systematic review is to review existing literature to determine the effect of balanced versus unbalanced fluid bolus therapy on metabolic acidosis in critically ill children.

**Methods:**

Using the Preferred Reporting Items for Systematic Review and Meta-analysis Protocols (PRISMA-P) guidelines, we will conduct a systematic review to retrieve all controlled trials and observational studies comparing balanced and unbalanced resuscitative fluids in critically ill children from age 28 days to 18 years old in any resuscitation settings. Search strategy was developed in collaboration with an experienced clinical research librarian. The primary outcome is the incidence and/or time to resolution of metabolic acidosis. Secondary outcomes included the incidence of hyperchloremia, AKI, duration of renal replacement therapy, vasopressors, mechanical ventilation, total volume of rehydration needed per day, extracorporeal membrane oxygenation, and length of stay and mortality. Study screening, inclusion, data extraction, and assessment of risk of bias will be performed independently by two authors. We intend to perform a meta-analysis with studies that are compatible on the basis of population and outcomes.

**Discussion:**

Isotonic crystalloid fluid bolus therapy is a ubiquitous treatment in resuscitation of critically ill pediatric patients and yet there is no clear recommendation to support the choice of balanced versus unbalanced fluid. The present review will summarize current available data in the literature and assess whether recommendations can be generated regarding the choice of crystalloids or otherwise identify knowledge gaps which will open the door to a large-scale randomized controlled trial (RCT).

**Electronic supplementary material:**

The online version of this article (10.1186/s13643-019-1109-2) contains supplementary material, which is available to authorized users.

## Background

Intravenous (IV) fluid bolus therapy is used in the resuscitation of critically ill children for numerous conditions including severe respiratory distress, dehydration, sepsis, diabetic ketoacidosis (DKA), and trauma [1, 2]. Goals of fluid in resuscitation are to replace intravascular volume and restore or maintain hemodynamic stability. Among numerous solutions available [see Additional file 1: Table S1], the ideal choice of isotonic crystalloid solution for fluid bolus therapy remains unclear despite being frequently used [3–7].

The most easily available and most frequently used crystalloid for fluid bolus therapy is 0.9% saline, an isotonic crystalloid composed of 154 mmol/L of sodium and 154 mmol/L of chloride, which is effective for intravascular volume restoration with minimal extravascular fluid retention [8]. However, 0.9% saline administration can lead to an increase in serum chloride concentration with subsequent decrease in the serum bicarbonate concentration, which may result in dilutional hyperchloremic metabolic acidosis. Hyperchloremia increases chloride concentration in the macula densa, which increases afferent arteriolar resistance thereby reducing renal blood flow and the glomerular filtration rate.

Balanced IV fluids were developed with the goal of being more physiologic with a fluid composition closer to the composition of serum, hence reducing the risk/incidence of hyperchloremic metabolic acidosis. Ringer’s lactate (RL), the most commonly used balanced solution, contains 130 mmol/L of sodium, 109 mmol/L of chloride, 28 mmol/L of lactate, 4 mmol/L of potassium, and 1.4 mmol/L of calcium. However, RL is more expensive (C$1.80 for 1 L of RL versus C$1.41 for 1 L of 0.9% saline) and less readily available than 0.9% saline. In addition, RL has been associated with increased serum lactate, which can confound its interpretation as a biomarker of tissue oxygenation [5]. The Federal Drug Administration (FDA) in the USA has highlighted potential adverse effects with RL such as hyperkalemia in patients with renal impairment, hyponatremia, and metabolic alkalosis. In addition, RL is incompatible with blood product and ceftriaxone administration due to the risk of coagulation and precipitation, respectively [9]. Furthermore, controversies exist regarding RL in patients with closed head injury as the solution has a lower osmolality than 0.9% saline, which might have an impact on cerebral edema [10, 11].

In vitro, when compared to other types of metabolic acidosis, hyperchloremic acidosis has been associated with NO release leading to pro-inflammatory cascade activation and a decreased glomerular filtration rate [12–14]. In the critically ill adult population, Semler reported an incidence of hyperchloremia, defined as chloride > 110, of 42.1% in the unbalanced group as opposed to 35.2% in the balanced group (*p* < 0.001) [15]. Although some trials are inconclusive, many have found that balanced fluids have been associated with less hyperchloremia acidosis, acute kidney injury (AKI), RRT, and mortality [15–19]. However, data in the critically ill children populations is limited. In non-critically ill children undergoing major surgery, balanced solutions have been associated with less hyperchloremia and less metabolic acidosis when compared to 0.9% saline [20]. Moreover, a randomized controlled trial (RCT) on initial IV fluid in children with DKA showed Hartmann’s solution, when compared to 0.9% saline, to be associated with shorter hospital length of stay overall and a shorter time to normalization of pH in the subgroup of severe DKA [21]. However, in pediatric acute severe diarrheal dehydration, Kartha et al. showed no difference between RL and 0.9% saline in biochemical or clinical outcomes [22] while Allen et al. showed more rapid improvement of serum bicarbonate and faster resolution of dehydration with RL [23]. In children with dengue fever, an RCT comparing several resuscitation fluids (Dextran, Gelatin, 0.9% saline, and RL) showed that hemodynamic recovery was prolonged in the RL group [24]. The current limited data makes it difficult to draw conclusions on the effect of unbalanced versus balanced fluid bolus therapy in critically ill children.

### Research question

This study aims to review the current literature to compare the effect of balanced versus unbalanced fluids bolus therapy on the incidence of metabolic acidosis in critically ill children.

## Methods and design

This protocol was designed and written according to the Preferred Reporting Items for Systematic Review and Meta-analysis Protocols (PRISMA-P) guidelines for reporting systematic reviews and will be registered in PROSPERO, an international prospective register of systematic reviews [see Additional file 2: PRISMA-P 2015 Checklist] [25–27].

### Data sources and search strategy

An electronic search strategy was developed in collaboration with an experienced clinical research librarian using a method designed to optimize term selection [28]. The following databases will be searched: MEDLINE including Epub Ahead of Print, In-Process & Other Non-Indexed Citations, and Embase and the CENTRAL Trials Registry of the Cochrane Collaboration using the Ovid interface [see Additional file 3: Search Strategy]. The search will include all published studies with no restriction of language or journal of publication. We will validate the search strategy to confirm appropriate literature coverage with the *Inquisitio Validus Index Medicus* validation method based on known item method [29, 30]. References of major studies, review articles, and included studies will be reviewed. Unpublished and ongoing trials will be identified using ClinicalTrials.gov and the WHO International Clinical Trials Registry Platform.

### Study designs

RCTs, quasi-randomized controlled trials, and observational cohort studies that evaluate the effect of administration of balanced versus unbalanced fluid bolus therapy on clinical outcomes will be eligible. Case-control, cross-sectional, case studies/series/report, reviews, and editorials will be excluded.

### Population

The population of interest is critically ill children, aged from 28 days to less than 18 years old with any severe problem with the airway, breathing, circulation, or acute deterioration of conscious state, as defined by the World Health Organization [31], that are requiring active fluids bolus therapy in any setting: emergency department, intensive care unit (ICU) (e.g., medical, surgical, cardiac, burn, or neurological), operating room, or inpatient step-down units. The study population is intentionally heterogeneous since the primary outcome, metabolic acidosis, has been shown to occur with unbalanced solutions in numerous conditions: surgical [20, 32], sepsis [33], DKA [34], gastroenteritis [23]. However, premature infants and neonates under 28 days of life were excluded because of their immature fluid and electrolyte homeostasis and their adaptive renal function [35].

### Intervention

Studies evaluating the impact of balanced versus unbalanced fluid bolus therapy on clinical outcomes will be included. Unbalanced fluids are defined as 0.9% saline, a sodium- and chloride-based solution with no added buffers. Balanced fluids are defined as sodium based with chloride content less than 154 mmol/L, allowing for other buffers to be added and better approximation of the plasma composition. They include, but are not limited to, Hartmann’s solution, Lactated Ringer’s solution, Ringer’s Acetate, Plasma-Lyte, Sterofundin, Ionosteril, and Isolyte. Fluid bolus therapy has to be administered with a minimal quantity of 20 cc/kg or 1 L cumulative within the first 72 h of hospital admission [36–38]. If the solution contains added dextrose, it will remain eligible as long as it is the appropriate isotonic crystalloid solution and respects the minimal volume required. Indeed, dextrose is not expected to interact with the effect of isotonic crystalloid solution. Route of administration may be IV, intra-osseous, or both. Studies assessing strictly maintenance fluids will be excluded.

### Comparator

Balanced fluids will be compared to 0.9% saline. Colloids will be excluded.

### Outcome

The primary outcome will be the incidence and/or time to resolution of metabolic acidosis defined as a serum pH < 7.35 or serum bicarbonates < 20, within 24 h of fluid bolus therapy. Secondary outcomes will be incidence of hyperchloremia defined as chloride > 106 mmol/L within 24 h of fluid bolus therapy, AKI as defined by pRIFLE or AKIN or KDIGO within 48 h of the fluid bolus therapy [39–41], duration of renal replacement therapy, duration of vasopressors, duration of mechanical ventilation, total volume of rehydration needed per day, need for extracorporeal membrane oxygenation (ECMO), ICU and hospital length of stay, and mortality. If composite outcomes are reported, we will report the component outcomes if available.

### Study screening and selection

A study screening form will be developed based on the inclusion and exclusion criteria for study screening and selection. Using Covidence software, two independent authors (SRD, AL) will determine eligibility of studies for inclusion by assessing titles and abstracts. At 10% of screening, conflict will be assessed and clarification will be added to the screening criteria if needed. All studies identified as eligible by at least one author will have full-text articles retrieved and assessed. Studies deemed eligible by both authors will be included in the systematic review. A kappa coefficient for interrater agreement will be reported. If consensus on inclusion of the study cannot be reached after full-text article screening, a third independent reviewer (KM) will resolve the disagreement. Reason for exclusion for all ineligible studies will be recorded. A synthesis of all studies excluded and included according to the search strategy will be presented using a PRISMA flow diagram.

### Data extraction

A standardized form summarizing all relevant data to be extracted from each included study is presented in Additional file 4: Data extraction form. Two authors (SRD, AL) will independently proceed with data extraction. A calibration test will occur with 10% of the included studies to optimize reliability in data extraction, and clarification will be added to the extraction form if needed. Disagreements will be resolved by consensus. Lack of agreement will be resolved by a third independent reviewer (KM). A kappa coefficient for interrater agreement will be reported. The following data on study characteristics will be collected: study design including intended outcomes, study period, population size, and demographics (age, gender, principal diagnosis, severity of illness, co-morbidities); inclusion and exclusion criteria; group differences; intervention details (type and quantity of fluid); co-interventions (blood products, steroids, dextrose, etc.); and setting (emergency department, intensive care unit, operating room, inpatient step-down/up units). Outcomes we will collect include the following: incidence and/or time to resolution of metabolic acidosis, hyperchloremia, AKI, renal replacement therapy, vasopressors, mechanical ventilation, total volume of rehydration needed per day, ECMO, ICU, and mortality. Source of funding will also be reported. If key information is missing regarding study data, the corresponding authors will be contacted up to two times to clarify. Imputation will be attempted only if statistically feasible.

### Risk of bias assessment

Two authors will independently assess the risk of bias for each included study. To assess the risk of bias of non-RCTs, we will use the ROBINS-I tool which covers seven domains: (1) bias due to confounding, (2) bias in selection of participants into the study, (3) bias in classification of interventions, (4) bias due to deviations from intended interventions, (5) bias due to missing data, (6) bias in measurement of outcomes, and (7) bias in selection of the reported results. The magnitude of the bias will be expressed as “low risk” if all domains are considered low risk of bias, “moderate risk” if all domains are considered low or moderate risk of bias, “serious risk” if one or more domains are considered serious risk of bias, “critical risk” if one or more domains are considered critical risk of bias, or “no information” if one or more domains have unclear risk of bias [42]. To assess the risk of bias of RCTs, we will use the Cochrane Collaboration’s tool which covers six domains: (1) selection bias, (2) performance bias, (3) detection bias, (4) attrition bias, (5) reporting bias, and (6) other bias [41]. The magnitude of the bias will be expressed as “low” if all domains are considered low risk of bias, “high” if one or more domains are considered high risk of bias, or “unclear” if one or more domains have unclear risk of bias. A risk can be unclear from missing details with inability to obtain clarification from the authors or because the impact of the bias on the measured outcome is uncertain [43]. We will also assess the direction of the bias.

### Evidence synthesis

Descriptive statistics will be provided on all included studies. Data on study characteristics, interventions, outcomes, and important covariates will be summarized using frequency and percentage for dichotomous outcomes, and means and standard deviation or median and inter-quartile range for continuous outcomes. For binary outcomes relative risk and number needed to treat (NNT) with 95% confidence interval will be used as an effect measure. For continuous outcomes, we will use mean difference or standardized mean difference (if units differ). Statistical significance will be determined at a level *α* ≤ 0.05. Heterogeneity among studies will be estimated using the *I*^2^ statistics, and we will elucidate heterogeneity by examining various sources of heterogeneity including patient populations, settings, and interventions. We will only pool estimates if the studies are considered comparable. Excluding non-RCT studies and high bias studies, we will perform random effects meta-analysis to pool results. If a meta-analysis is not appropriate, a qualitative synthesis of findings will be presented. All analysis will be performed using the R statistical software.

### Subgroup and sensitivity analysis

If feasible, subgroup analysis will be performed as follows: (1) age < 1 year old versus > 1 year old, (2) surgical versus non-surgical, and (3) 20–40 cc/kg versus > 40 cc/kg of fluid bolus therapy. Children under the age of 1 year tend to have different physiology and different etiology of illness. Surgical patients are exposed to specific pathophysiology due to the use of anesthetics and a possible inflammatory response to the surgery. Analyzing the subgroup of high volume of fluid bolus therapy could increase the effect size and therefore demonstrate more differences between groups.

### Summary and dissemination of findings

The Grading of Recommendations Assessment, Development and Evaluation (GRADE) approach will be used to assess the quality of evidence for the primary and secondary outcomes. The findings of the present systematic review will be published in a peer-reviewed journal and presented at different conferences and scientific meetings. The findings will be used to design a future large-scale RCT of balanced versus unbalanced fluids bolus therapy in critically ill children.

## Discussion

Fluid bolus therapy is a ubiquitous treatment in the resuscitation of critically ill children and yet there is no clear guideline recommendation to support the choice of balanced versus unbalanced fluid. The present systematic review will summarize the available data in the current literature to assess whether recommendations can be generated regarding the choice of crystalloids. Alternatively, it will identify knowledge gaps, which will open the door to a large-scale RCT.

A RCT comparing balanced versus unbalanced fluids in the critically ill pediatric population would have two possible outcomes both of which would significantly influence clinical practice. If the RCT demonstrates clinical benefits of balanced over unbalanced fluids, it would lead to official recommendations on the choice of fluid bolus therapy in this population. However, if no clinical benefits of balanced fluids can be established, it would imply that its use as a first-line agent is no longer justified, as they are more expensive and less accessible. Therefore, no matter the outcome, it will standardize practice and have an impact on patient care and resource utilization in the health care system.

## Additional files

Additional file 1: : Table S1 Type and composition of different isotonic crystalloids solution compared to human plasma. (PDF 54 kb)

Additional file 2: PRISMA-P 2015 Checklist (PDF 159 kb)

Additional file 3: Search strategy (PDF 54 kb)

Additional file 4: Data extraction form (PDF 61 kb)

## Data Availability

Not applicable.

## Abbreviations

AKI: Acute kidney injury
DKA: Diabetic ketoacidosis
ECMO: Extracorporeal membrane oxygenation
FDA: Federal Drug Administration
GRADE: Grading of Recommendations Assessment, Development and Evaluation
ICU: Intensive care unit
IV: Intravenous
PRISMA-P: Preferred Reporting Items for Systematic Review and Meta-analysis Protocols
RCT: Randomized controlled trials
RL: Ringer’s lactate
