# Supplementary material for: Impact of balanced versus unbalanced fluid resuscitation on clinical outcomes in critically ill children: protocol for a systematic review and meta-analysis
Source: Syst Rev. 2019 Aug 5;8:195. doi: 10.1186/s13643-019-1109-2 (PMC6683512; doi:10.1186/s13643-019-1109-2)
Supplement: Supplementary file 1 — : Table S1 Type and composition of different isotonic crystalloids solution compared to human plasma. (PDF 54 kb) [file 13643_2019_1109_MOESM1_ESM.pdf]

Table 1. Type and composition of different isotonic crystalloids solution compared to human plasma

	Human plasma	Crystalloids							
		0.9% NaCl	Compound Sodium Lactate	Ringer's Lactate	Ringer's Acetate	PlasmaLyte	Sterofundin	Ionosteril	Isolyte
Osmolarity (mOsm/L)	291.0	308.0	278.0	272.0	276.0	294.0	309.0	291.0	295.0
pH	7.35-7.45	5.5	5.0-7.0	6.5	6-8	7.4	5.1-5.9	6.9-7.9	6.7
Na (mmol/L)	140.0	154.0	129.0	130.0	130.0	140.0	145.0	137.0	140.0
Potassium (mmol/L)	4.5	-	5.0	4.0	5.0	5.0	4.0	4.0	5.0
Calcium (mmol/L)	2.4	-	2.0	1.4	1.0	-	2.5	1.7	
Magnesium (mmol/L)	0.9	-	-	-	1.0	1.5	1.0	1.3	3.0
Chloride (mmol/L)	100.0	154.0	109.0	109.0	112.0	98.0	127.0	110.0	98.0
Bicarbonate (mmol/L)	24.0	-	-	-	-	-		-	
Lactate (mmol/L)	1.5	-	29.0	28.0	-	-		-	
Acetate (mmol/L)	-	-	-	-	27.0	27.0	24.0	36.8	27.0
Malate (mmol/L)	-	-	-	-	-	-	5.0	-	
Gluconate (mmol/L)		-	-	-	-	23.0		-	23.0
