# Supplementary material for: Impact of balanced versus unbalanced fluid resuscitation on clinical outcomes in critically ill children: protocol for a systematic review and meta-analysis
Source: Syst Rev. 2019 Aug 5;8:195. doi: 10.1186/s13643-019-1109-2 (PMC6683512; doi:10.1186/s13643-019-1109-2)
Supplement: Supplementary file 3 — Search strategy (PDF 54 kb) [file 13643_2019_1109_MOESM3_ESM.pdf]

Search Strategy

Searches were conducted using an Ovid multi-database search and duplicate records were removed online giving preference to MEDLINE, then Embase, with no field preference. Lines 1-5 are optimized for MEDLINE and the main question constructs are broken out in separate lines for clarity. Lines 6-11 are optimized for Embase and line 12 is optimized for CENTRAL. The next lines isolate the records to the database the search was designed for, combine those sets and then remove duplicate records and final isolate the records from each database again so each can be downloaded and imported into the citation manager using a database-specific import filter.

1 Isotonic solutions/ or (plasma-lyte.mp. or plasmalyte or Ringer* or low chloride or sterofundin or ionosteril or isolyte).ti,ab,kf. or ((hartman* or balance* or buffered or lactat*) adj2 (crystalloid or solution* or saline)).ti,ab,kf.
2 exp Sodium Chloride/ or (Saline or (NaCl adj "0.9")).ti,ab,kf.
3 exp Fluid Therapy/ or Critical Illness/ or exp Sepsis/ or exp Shock, Traumatic/ or exp Intensive Care Units/ or exp Hospitalization/ or Inpatients/ or Child, Hospitalized/ or Adolescent, Hospitalized/ or Emergencies/ or exp Emergency Medical Services/ or Emergency Medicine/ or exp Emergency Treatment/ or (emergenc* or ED or casualty department*).ti,ab,kf. or (resus* or haemodynamic* or hemodynamic* or intensive care or PICU or NICU or ICU).mp.
4 (child* or adolescent* or infan*).mp.
5 1 and 2 and 3 and 4
6 Hartmann Solution/ or Ringer Acetate/ or Ringer Glucose Solution/ or Ringer Lactate Solution/ or Ringer Solution/ or ((Crystalloid/ or Infusion Fluid/) and (balance* or physiolog* or buffer*).ti,ab,kw.) or plasma-lyte.mp. or (plasmalyte or Ringer* or low chloride or sterofundin or ionosteril or isolyte).ti,ab,kw. or ((hartman* or balance* or buffered or lactat*) adj2 (crystalloid or solution* or saline)).ti,ab,kw.
7 exp Sodium Chloride/ or (Saline or (NaCl adj "0.9")).ti,ab,kw.
8 exp Fluid Resuscitation/ or Critical Illness/ or exp Sepsis/ or exp Shock/ or exp Intensive Care Unit/ or exp Hospitalization/ or exp Child hospitalization/ or Emergency Ward/ or Emergency Treatment/ or Emergency Care/ or Emergency Health Service/ or Emergency Medicine/ or Emergency/ or (emergenc* or ED or casualty department* or resus* or haemodynamic* or hemodynamic* or intensive care or PICU or NICU or ICU).ti,ab,kw.
9 6 and 7 and 8
10 9 and (baby or babies or infan* or preschool* or pre-school* or child* or pediatr* or paediatr* or teen* or adolescen*).mp.
11 limit 10 to embase [Limit not valid in Ovid MEDLINE(R),Ovid MEDLINE(R) Daily Update,Ovid MEDLINE(R) In-Process,Ovid MEDLINE(R) Publisher; records were retained]
12 ((plasma-lyte or plasmalyte or Ringer* or low chloride or sterofundin or ionosteril or isolyte or ((hartman* or balance* or buffered or lactat*) adj2 (crystalloid or solution* or saline))) and (Saline or (NaCl adj "0.9"))).ti,ab,kw. and (resus* or haemodynamic* or hemodynamic* or intensive care or PICU or NICU or ICU or emergenc* or ED or

casualty department*).mp. and (baby or babies or infan* or preschool* or pre-school* or child* or pediatr* or paediatr* or teen* or adolescen*).mp.

13	5 use medall
14	11 use emczd
15	12 use cctr
16	or/13-15
17	remove duplicates from 16
18	17 use medall
19	17 use emczd
20	17 use cctr

ClinicalTrials.gov seach
https://clinicaltrials.gov/ct2/results?cond=resuscitation&term=fluid&type=&rslt=&age_v=&age=0&gndr=&intr=&titles=&outc=&spons=&lead=&id=&cntry=&state=&city=&dist=&locn=&strd_s=&strd_e=&prcd_s=&prcd_e=&sfpd_s=&sfpd_e=&lupd_s=&lupd_e=

WHO ICTRP
fluid and resuscitation (limited to clinical trials in children)
