# Supplementary material for: Impact of balanced versus unbalanced fluid resuscitation on clinical outcomes in critically ill children: protocol for a systematic review and meta-analysis
Source: Syst Rev. 2019 Aug 5;8:195. doi: 10.1186/s13643-019-1109-2 (PMC6683512; doi:10.1186/s13643-019-1109-2)
Supplement: Supplementary file 4 — Data extraction form (PDF 61 kb) [file 13643_2019_1109_MOESM4_ESM.pdf]

Data extraction form

Identification

- Title of the study
- Source of funding
- Country
- Journal and year of publication
- Authors

Methodology

- Design
- Study period
- Intended outcome
- Other relevant details

Population

- Size (included/enrolled)
- Age
- Gender
- Principal diagnosis
- Severity of illness
- Co-morbidities
- Inclusion criteria
- Exclusion criteria
- Group differences
- Hospital setting (emergency department, intensive care unit, operating room, inpatient step-down/up units)

Intervention

- Type fluid
- Quantity of fluid
- Co-interventions (such as blood, steroid, dextrose…)
- Other relevant details

Outcome

- Incidence and/or time to resolution of metabolic acidosis
- Hyperchloremia
- AKI
- Renal replacement therapy
- Vasopressors
- Mechanical ventilation
- Total volume of rehydration needed per day
- ECMO
- ICU length of stay

- Hospital length of stay
- Mortality

Overall conclusion
